# A randomized, prospective, masked clinical trial comparing an opioid-free vs. opioid-sparing anesthetic technique in adult cats undergoing ovariohysterectomy

**DOI:** 10.3389/fvets.2022.1002407

**Published:** 2022-11-11

**Authors:** Maxime Rufiange, Helene L. M. Ruel, Beatriz P. Monteiro, Ryota Watanabe, Inga-Catalina Cruz Benedetti, Javier Benito, Paulo V. M. Steagall

**Affiliations:** ^1^Department of Clinical Sciences, Faculty of Veterinary Medicine, Université de Montréal, Saint-Hyacinthe, QC, Canada; ^2^Department of Veterinary Clinical Sciences and Centre for Companion Animal Health and Welfare, Jockey Club College of Veterinary Medicine and Life Sciences, City University of Hong Kong, Kowloon, Hong Kong SAR, China

**Keywords:** analgesia, animal welfare, feline, opioid-free, opioid-sparing, pain, population control, kittens

## Abstract

This study aimed to compare the analgesic effects of an injectable protocol using multimodal analgesia with or without opioids in cats undergoing ovariohysterectomy (OVH). Thirty-two healthy cats were enrolled in a prospective, blinded, randomized trial after the caregiver's written consent. Cats received a combination of ketamine (4 mg/kg), midazolam (0.25 mg/kg) and dexmedetomidine (40 μg/kg), and either buprenorphine (20 μg/kg) or saline (same volume as buprenorphine) intramuscularly [opioid-sparing (OSA) and opioid-free anesthesia (OFA) groups, respectively]. Intraperitoneal bupivacaine 0.25% (2 mg/kg) and meloxicam (0.2 mg/kg subcutaneously) were administered before OVH. Atipamezole (400 μg/kg intramuscularly) was administered at the end of surgery. Pain and sedation were evaluated using the Feline Grimace Scale (FGS) and a dynamic interactive visual analog scale, respectively. Intravenous buprenorphine was administered as rescue analgesia if FGS scores ≥ 0.39/1. Statistical analysis included repeated measures linear mixed models, Fisher's exact test and Bonferroni adjustments when appropriate (*p* < 0.05). Twenty-seven cats were included. The prevalence of rescue analgesia was lower in OSA (*n* = 0/13) than in OFA (*n* = 5/14) (*p* = 0.04). The FGS scores (least square means and 95% CI) were higher in OFA at 1 [2.0 (1.3–2.7)] and 2 h [2.2 (1.5–2.9)] than baseline [0.7 (0.0–1.4)], but not in OSA. Sedation scores were not significantly different between groups. Opioid-free injectable anesthesia was appropriate for some cats using a multimodal approach. However, a single dose of intramuscular buprenorphine eliminated the need for rescue analgesia and assured adequate pain management after OVH in cats.

## Introduction

Opioids are the cornerstone of acute pain management (1). They are included in the World Small Animal Veterinary Association list of essential medicines for cats and dogs as core medicines (2). However, veterinarians do not always have access to these analgesic drugs in many countries, as it often occurs with inhalant anesthetics. For example, spay-neuter programs are often performed with injectable anesthetics with limited or no perioperative analgesia in several countries (3); the efficacy of these analgesic protocols are unknown and a large number of animals may be painful postoperatively. Furthermore, opioid drug shortages have recently become a problem with the opioid epidemic, a public health emergency (4), that also impacted the veterinary profession and affected the ability of veterinarians in the treatment of animal pain. Indeed, veterinarians may struggle to find reasonable options to manage pain effectively without opioids (5).

Opioid-free anesthesia (OFA) is a practice that excludes perioperative administration of opioids and uses non-opioid, multimodal analgesic techniques. Opioid-sparing anesthesia (OSA) is a technique where small amounts of opioids are used intraoperatively (i.e., single and/or low doses of perioperative opioids) (6, 7). The opioid epidemic has fueled the study of OFA and OSA as a new area of research in human medicine to help avoid or reduce opioid-induced adverse effects, including respiratory depression and death (6, 8, 9). As much as opioid-induced adverse effects are different between humans and cats, the study of OFA is fundamental in feline medicine to overcome any future opioid shortages. There is also an urgent need to provide humane alternatives for pain management in sterilization programs, as analgesia is often suboptimal (3).

Studies on OFA and alternatives to inhalant anesthesia are lacking in veterinary medicine (10–16). In a previous study performed in our laboratory, a protocol with an intramuscular (IM) injection of ketamine-dexmedetomidine-midazolam (KET-DEX-MID) provided wide safety margin, rapid immobilization, and adequate duration and depth of anesthesia in cats undergoing ovariohysterectomy (OVH) using non-opioid analgesic techniques (14). However, the study protocol did not provide optimal postoperative analgesia in more than half of the studied population. A major limitation of that study was the lack of direct comparisons between OFA and OSA and the study population including both kittens and adult cats. In the present study, we investigated the use of KET-DEX-MID IM with (OSA) or without (OFA) an opioid (buprenorphine) using a multimodal analgesic regimen including intraperitoneal administration of a local anesthetic (bupivacaine) and a nonsteroidal anti-inflammatory drug (meloxicam) in cats older than 6 months of age.

The hypothesis of this study was that OFA would have a higher prevalence of rescue analgesia and postoperative pain scores than OSA using multimodal analgesia in cats undergoing OVH. The objective of the study was to compare the analgesic effects of KET-DEX-MID using multimodal analgesia with (OSA) or without buprenorphine (OFA) in cats undergoing OVH.

## Materials and methods

The study protocol was approved by the animal care committee of the Faculty of Veterinary Medicine (FMV), Université de Montréal (20-RECH-2075). The study is reported according to the Consolidated Standards of Reporting Trials (CONSORT guidelines) (17).

### Animals

Thirty-two female cats from local shelter facilities were enrolled in a prospective, randomized, blinded (masked) clinical trial after obtaining the caregivers' (shelter) written consent. Inclusion criteria included any healthy cat of any breed older than 6 months of age. Cats were considered healthy based on medical history, physical examination, up-to-date vaccination and deworming status, and hematocrit and total protein values. Exclusion criteria included body weight <1.5 kg, cardiac dysrhythmias on auscultation, body condition score ≥7 or ≤3 (scale 1–9), shy or fearful individuals not allowing preoperative pain assessment, preoperative pain scores consistent with presence of mild pain (Feline Grimace Scale; FGS scores of ≥3/10) and visualization of scar/tattoo as evidence of previous OVH. Cats were admitted ~16 h before general anesthesia and housed individually in cages located in a cat ward. Cages included water, food, a toy, a card box, a blanket and a litter box.

### Treatment randomization

Cats were randomized in one block with allocation ratio of 1:1. Randomization was performed by an individual not involved with pain assessment (RW) using a randomization plan generator (www.randomization.com). Each cat was assigned a number (1–32) upon arrival. According to this number, the patient was allocated to one of the two (OFA or OSA) treatment groups (*n* = 16/group). If any cat was excluded, additional cats were recruited and given the same treatment as the excluded cat.

### Anesthesia, analgesia and surgery

Food, but not water was withheld for ~6–8 h before the administration of anesthetics. The anesthetic protocol included a single injection with a combination of ketamine (4 mg/kg; Ketaset, Zoetis, Kirkland, Quebec), dexmedetomidine (40 μg/kg; Dexdomitor, Zoetis, Kirkland, Quebec) and midazolam (0.25 mg/kg; Sandoz, Boucherville, Quebec) (KET-DEX-MID) administered into the lumbar muscles by individuals not involved with pain assessment (HLMR, JB, and PVS) (14). Drugs were withdrawn in the following order: ketamine, dexmedetomidine and midazolam. Additionally, cats received either IM buprenorphine (20 μg/kg; Vetergesic, Champion Alstoe, Whitby, Ontario; OSA) or saline (equivalent volume of buprenorphine; OFA) with KET-DEX-MID (*n* = 16/group). Buprenorphine or saline were added to the KET-DEX-MID solution.

After lateral recumbency was achieved, a 22-gauge catheter was aseptically inserted into the cephalic vein and the blood from the catheter was used to evaluate hematocrit and total protein. The cat was positioned in sternal recumbency and intubated with a supraglottic airway device (V-gel^®^ Docsinnovent, London, UK) connected to a capnograph; their eyes were also lubricated with ocular gel. Cats were allowed to breathe room air, unless oxygenation was required according to protocol below. Patients were then transferred to the operating room and placed in dorsal recumbency on a circulating warm water blanket for preparation of the surgical site.

Anesthesia was performed by the same veterinarian (RW). Physiologic variables were monitored using a multi-parametric monitor (LifeWindow 6,000V; Digicare Animal Health, Boynton Beach, FL, USA) and included arterial oxyhemoglobin saturation, end-tidal concentrations of carbon dioxide, respiratory rate, heart rate, systolic, mean and diastolic arterial pressure and temperature. Lactated Ringer's solution (Inj. Bag/500 mL, McCarthy and Sons Service, Calgary, Alberta) was administered intravenously at 5–10 mL/kg/h throughout the procedure and according to the patient needs.

Subcutaneous meloxicam (0.2 mg/kg; Metacam, Boehringer Ingelheim, Burlington, Ontario) was administered to all cats after anesthetic induction and before surgery; cats received a second dose orally (0.05 mg/kg; Metacam oral suspension; Boehringer Ingelheim, Burlington, Ontario) 24 h after the first dose. Ovariohysterectomy was performed using a ventral midline incision and the feline pedicle tie technique by one experienced veterinarian (BPM) (18, 19). Bupivacaine hydrochloride 0.25% (2 mg/kg; Sensorcaine, AstraZeneca, Mississauga, Ontario) was administered by the intraperitoneal route using aseptic technique, as previously reported, after laparotomy and before OVH (20–22). The abdominal wall was closed using a simple continuous pattern and the skin and subcutaneous tissues using a continuous intradermal pattern. At the end of surgery, a 2-cm green tattoo was performed laterally to the incision for identification of a neutered cat. The surgical incision length (centimeters) was measured using a plastic rule. The protocol included the intravenous administration of ketamine (0.5 mg/kg) if signs of inadequate depth of anesthesia was observed (swallowing reflex, purposeful movement or sudden increases in MAP of ≥20% compared with pre-surgical values).

Extubation was done at the end of surgery, when palpebral reflexes returned. Atipamezole (400 μg/kg IM; Antisedan, Zoetis, Kirkland, Quebec) was administered 15 min after the end of surgery by an individual dedicated to the anesthetic recovery (ICCB). The following parameters were recorded for each cat: onset of anesthesia (time from the end of IM injection KET-DEX-MID until lateral recumbency using a chronometer), duration of surgery (time from first incision to placement of the last suture), duration of anesthesia (time from KET-DEX-MID injection to atipamezole injection), time to sternal recumbency (time from atipamezole injection to first sternal recumbency) and head lift (time from atipamezole injection to first head lift).

### Pain and sedation assessment

Pain assessment was performed in real-time using the FGS at least 4 h after patient admission (time 0, baseline) and at 0.5, 1, 2, 4, 6, 8, and 24 h after the end of surgery by the same veterinarian (MR) who was blinded to the treatment groups and not involved with drug preparation or administration. Training on pain assessment was not provided before the study had begun, but this individual was a final year resident of a program registered with the American College of Veterinary Anesthesia and Analgesia with previous experience in clinical feline pain evaluation. The FGS is a facial expression-based scoring system consisting of five action units (ear position, orbital tightening, muzzle tension, whiskers change and head position; each scored from 0 to 2) (23, 24). For pain scoring, the cat was examined in the cage without being disturbed. Rescue analgesia was administered using buprenorphine (20 μg/kg IV) if pain scores were ≥0.39/1 using the FGS (24). Therefore, the FGS was recorded as the total score divided by the maximum scores using only the action units included in the evaluation. Pain assessment continued for all cats until the end of the study.

After pain scoring, sedation was evaluated by the same individual (MR) using a dynamic interactive visual analog scale (DIVAS) at the same time points described above. The DIVAS score was derived from a 10 cm line where 0 corresponds to “no sedation” and 10 corresponds to the “maximum sedation possible”.

Anesthetic complications were defined as: tachycardia (heart rate > 160 bpm), bradycardia (heart rate < 70 bpm), hypotension (mean arterial pressure < 50 mmHg), bradypnea (respiratory rate < 4 rpm), tachypnea (respiratory rate > 30 rpm), hypercapnia (end-tidal carbon dioxide concentrations > 50 mmHg), desaturation (oxygen saturation using pulse oximetry < 90%) and hypothermia (temperature < 36.0°C). Assisted ventilation using a manual resuscitator was performed in cases of hypercapnia or desaturation with duration of 5 min or more. If not resolved, 100% oxygen was provided by connecting a Mapleson D circuit to the supraglottic airway device.

### Statistical analysis

A data scientist with experience in statistics was consulted for power analysis. The prevalence of rescue analgesia was considered as the primary outcome because it provides important information on the decision-making process of painful cats in the clinical setting. In cats undergoing OVH, the prevalence of rescue analgesia was 57% using opioid-free anesthesia (14) and 6% when using an opioid-based regimen (25) in the context of multimodal analgesia in our laboratory. A power analysis with the Fisher's exact test indicated that a sample size of 12 would be needed in each group to detect such a difference in 80% of the times with the α level at 5%. Our clinical trials normally present an exclusion rate of ~25% (e.g., development of upper respiratory tract infection postoperatively, etc.) so the sample size was increased to 32 cats (*n* = 16/group) to account for potential exclusions and to minimize type II error.

Data were analyzed using R software within the integrated RStudio environment (versions 4.0.3 and 3.6.2, RStudio, Inc; RStudio Team (2020). RStudio: Integrated Development for R. RStudio, PBC, Boston, MA URL http://www.rstudio.com/). For all analysis, α was set at 5%. Data normality was assessed using the Shapiro-Wilk test. The prevalence of rescue analgesia was compared using the Fisher's exact test. The demographic characteristics of each group was investigated to assess differences between groups. Variables that were normally distributed (body weight, hematocrit, onset of anesthesia, duration of anesthesia, time to sternal recumbency and time to lift head) were compared between groups using two-tailed independent *t*-tests whereas those not normally distributed (age, body condition score, total protein and duration of surgery) were analyzed using two-tailed Wilcoxon test. The FGS and DIVAS sedation scores were analyzed using linear mixed models in which the best model was identified according to Bayesian information criterion. Time, group and their interaction were considered fixed effects. Cat nested within group was considered a random effect. Age was added as a co-variate to the model. The Bonferroni were used for adjustment after multiple comparisons.

## Results

**Figure 1:**
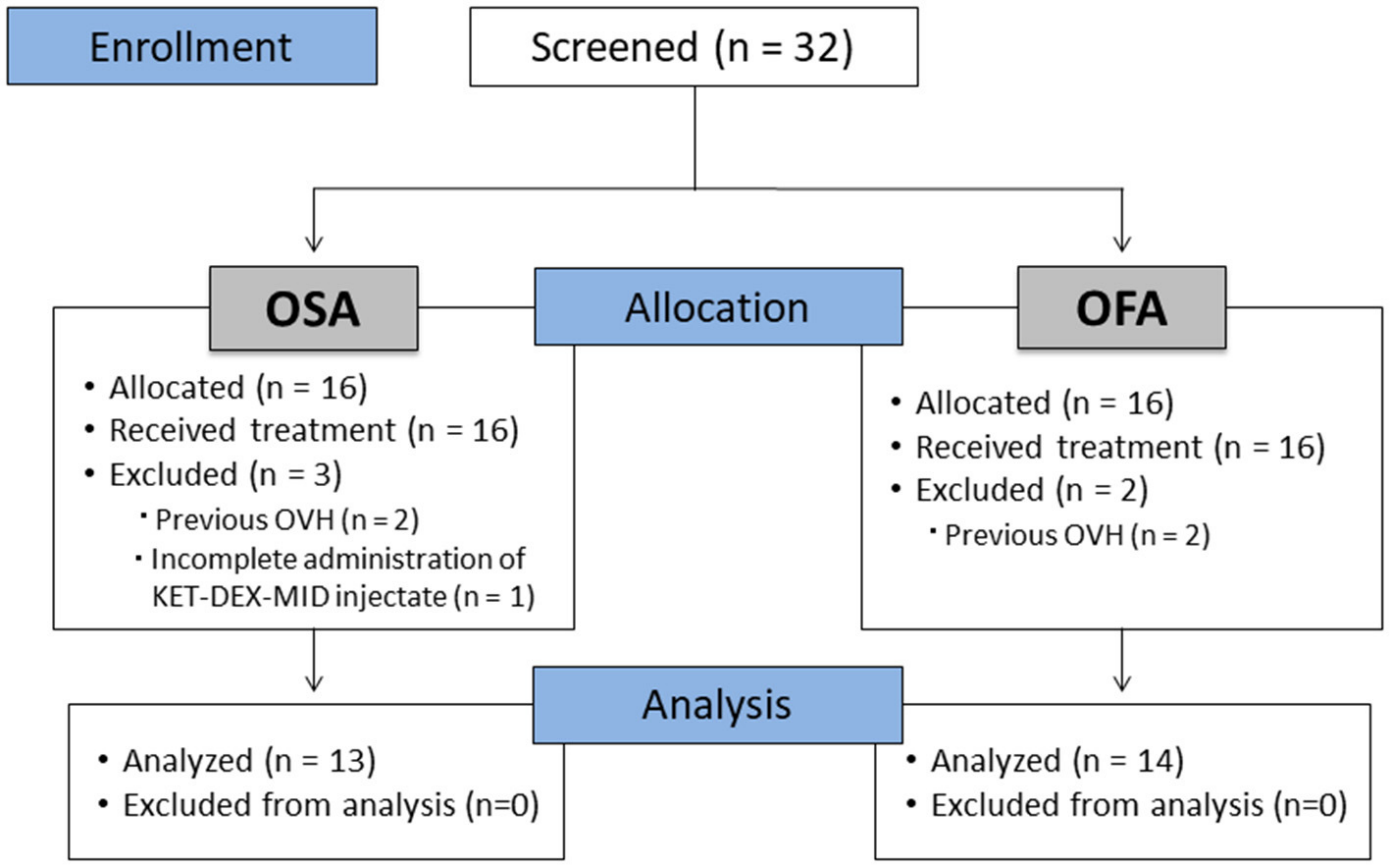
CONSORT flow diagram. OFA: Opioid-free anesthesia group; OSA: Opioid-sparing anesthesia group; OVH: Ovariohysterectomy.

Thirty-two cats were enrolled in the study (*n* = 16/group). Five cats were excluded (*n* = 2 in OFA that were already spayed and *n* = 3 in OSA - one cat did not receive the full volume of KET-DEX-MID and two were already spayed). Thus, a total of 27 cats completed the study (Figure 1). All cats were discharged at least 24 h after surgery without any complications.

Patient demographics, hematocrit, total protein, onset of anesthesia, duration of anesthesia and surgery, surgical incision length, and time to sternal recumbency were not different between the two treatment groups (Table 1). Time to lift head was significantly faster in the OFA compared with the OSA (*p* = 0.01, Table 1). None of the cats required the administration of ketamine during surgery. Two cats, one from each group, were in the mid-stages of pregnancy. Desaturation was observed in two cats in OSA. In one case, desaturation was resolved using a manual resuscitator. The other case required oxygenation. Tachypnea was observed in four cats (two cats in OFA and two cats in OSA). No other anesthetic complications were observed during the study.

**Table 1:** Population characteristics of cats undergoing ovariohysterectomy after the administration of buprenorphine (OSA) or saline (OFA) by the intramuscular route in combination with ketamine-dexmedetomidine-midazolam and multimodal analgesia.

	**OSA**	**OFA**	***p* value**
Age (years)	1 (0.65; 1.75)	1 (0.55; 2.5)	1.00
Body condition score	5 (4,5)	4.5 (4,5)	0.50
Total protein (g/L)	65 (60; 72)	61 (55; 70)	0.15
Hematocrit (%)	40 ± 5	41 ± 4	0.69
Duration of surgery (min)	17 (14; 19.5)	14 (14; 17.25)	0.27
Surgical incision length (cm)	2.3 (1.95; 2.65)	1.9 (1.7; 2.35)	0.11
Body weight (kg)	3.07 ± 0.59	2.72 ± 0.54	0.20
Onset of anesthesia (sec)	151 ± 47	126 ± 49	0.29
Duration of anesthesia (min)	50 ± 4	47 ± 3	0.10
Time to sternal recumbency (min)	11 ± 3	8 ± 3	0.15
Time to lift head (min)	8.2 ± 1.8^a^	5.6 ± 2.1^b^	0.01

Data are reported as median (interquartile interval) or mean ± standard deviation as appropriate. Different superscript letters indicate a significant difference between groups.

### FGS pain scores

**Figure 2:**
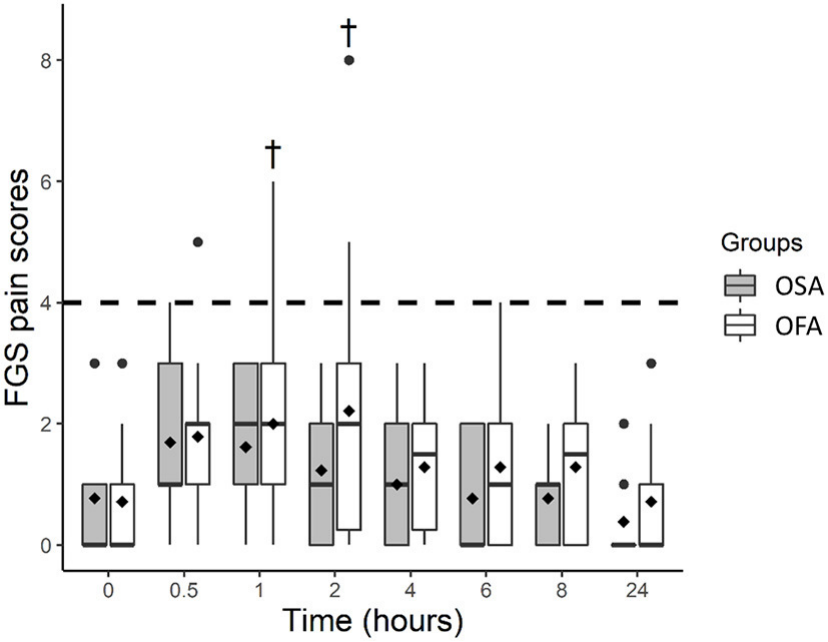
Boxplot of the Feline Grimace Scale (FGS) scores before (0 h) and at several time-points after ovariohysterectomy in cats receiving buprenorphine (OSA) or saline (OFA) by the intramuscular route in combination with ketamine-dexmedetomidine-midazolam and multimodal analgesia. • indicates outliers; ♦ indicates mean; † indicates a significant difference when compared with baseline values (0 h); the dotted line indicates the analgesic threshold of the FGS.

The FGS scores (least square means and 95% CI) were higher in OFA at 1 [2.0 (1.3–2.7)] and 2 h [2.2 (1.5–2.9)] than baseline [0.7 (0.0–1.4)], but not in OSA (Figure 2). The FGS scores were not significantly different between groups at any time point.

### Rescue analgesia

The prevalence of rescue analgesia was lower in OSA (*n* = 0/13) than in OFA (*n* = 5/14) (*p* = 0.04). Rescue analgesia was administered at 1 (*n* = 1), 2 (*n* = 2), 6 (*n* = 1), and 8 h (*n* = 1). None of the cats required more than one administration of rescue analgesia.

### Sedation scores

**Figure 3:**
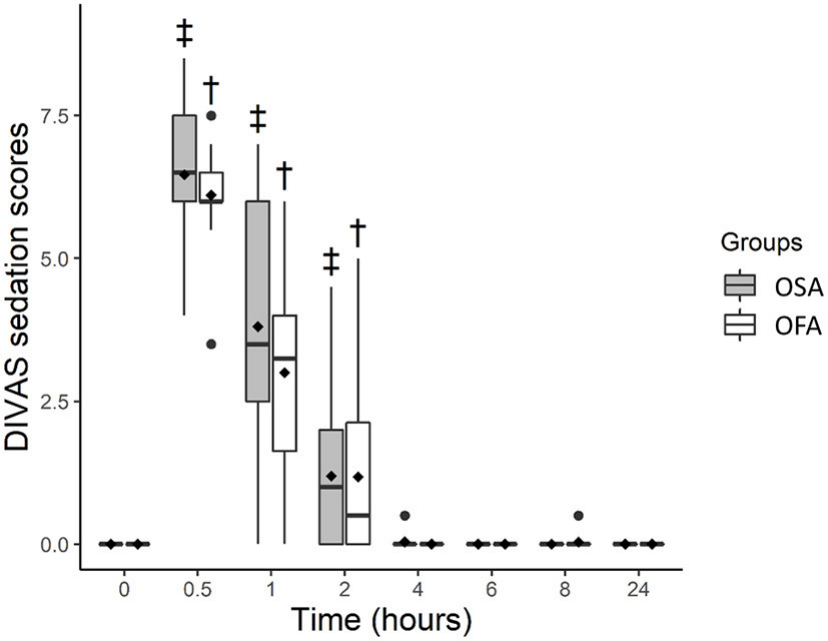
Boxplot of dynamic and interactive visual analog scale (DIVAS) sedation scores before (0 h) and at several time-points after ovariohysterectomy in cats receiving buprenorphine (OSA) or saline (OFA) by the intramuscular route in combination with ketamine-dexmedetomidine-midazolam and multimodal analgesia. • indicates outliers; ♦ indicates mean; † indicates significant difference in OFA when compared with baseline; ‡ indicates significant difference in OSA when compared with baseline.

DIVAS sedation scores were higher in OFA and OSA at 0.5 (*p* < 0.0001 for both), 1 (*p* < 0.0001 for both), and 2 h (*p* = 0.028 and *p* = 0.038, respectively) when compared with baseline values. Least square means and 95% confidence intervals were: OFA [baseline; 0.01 (−0.49–0.51)], [0.5 h; 6.12 (5.62–6.62), [1 h; 3.01 (2.5–3.5)], [2 h; 1.19 (0.69–1.69)] and OSA [baseline; −0.01 (−0.53–0.50)], [0.5 h; 6.45 (5.93–6.96)], [1 h; 3.79 (3.28–4.31)] and [2 h; 1.18 (0.66–1.70)]. Sedation scores were not significantly different between groups (Figure 3).

## Discussion

This study demonstrated that the use of an OSA technique with a single dose of intramuscular buprenorphine eliminated the need for postoperative rescue analgesia in cats undergoing OVH using KET-DEX-MID, intraperitoneal bupivacaine and preoperative administration of subcutaneous meloxicam. The hypothesis of the study was partially confirmed as a significant difference between OSA and OFA was observed for the prevalence of rescue analgesia. The FGS scores were not significantly different between groups but changes in relation to baseline were observed in OFA, but not in OSA. In brief, OFA anesthesia did not provide adequate analgesia using multimodal analgesia with intraperitoneal bupivacaine and subcutaneous meloxicam in all cats undergoing OVH. This finding demonstrates that, besides the advent of several non-opioid analgesic techniques, we still rely on the use of opioid analgesics for optimal perioperative pain management when combined with bupivacaine and meloxicam.

The study also provides novel information on OFA in cats. To the author's knowledge, there are only two publications addressing OFA in cats; one from our research group (20) and another study investigating OFA for orchiectomy (13). In our present study, the use of opioid-free injectable anesthesia was appropriate for the majority of cats of that group (*n* = 9/14) demonstrating that the multimodal approach with intraperitoneal bupivacaine and a nonsteroidal anti-inflammatory drug must be incorporated in routine OVH to reduce the prevalence of pain when opioids are not available. However, our study design herein could not confirm the superiority of using OFA with KET-DEX-MID with vs. without multimodal analgesia. Nevertheless, results of our laboratory have now confirmed that the use of non-opioid, multimodal analgesic techniques with intraperitoneal bupivacaine and preoperative administration of meloxicam provide lower prevalence of rescue analgesia than using KET-DEX-MID alone in kittens undergoing OVH (26). It is possible that in the future the addition of other non-opioid analgesic techniques could lead to a refinement and improvement in the efficacy of OFA protocols; for example, incisional in addition to intraperitoneal anesthesia with local anesthetics (20–22, 25, 27) and/or the administration of gabapentin (28).

Our results also support opioid accessibility as a core medicine in the veterinary profession as suggested by the World Small Animal Veterinary Association list of essential medicines for cats and dogs, particularly in sterilization programs (2). Our findings show that some cats would be unnecessarily painful after surgery even when other non-opioid, non-controlled analgesic techniques were used. It reaffirms that opioids are the cornerstone of acute pain management and veterinarians must have access to these analgesic drugs in order to provide optimal veterinary care.

This current methodology addressed some limitations of a previous study performed in our laboratory. In that clinical trial, we investigated two different doses of ketamine in combination with dexmedetomidine and midazolam using intraperitoneal administration of bupivacaine and meloxicam in cats undergoing OVH (14). The protocol provided rapid immobilization, unconsciousness and smooth anesthetic induction. The duration and depth of anesthesia were adequate and additional boluses of ketamine were not required during surgery using either 5 or 7 mg/kg of ketamine. Therefore, the anesthetic effects of KET-DEX-MID are in accordance to the Association of Shelter Veterinarians' 2016 guidelines for spay-neuter programs (29). However, the prevalence of rescue analgesia was ~57% of the population studied with significantly lower requirements in kittens (2/8) than adult cats (11/15) (14). Analgesia was considered inadequate even when other non-opioid analgesic techniques were administered. The main limitation of the previous clinical trial was that the design did not compare OSA vs. OFA and the study population involved both kittens and adults. In the current methodology, the study design allowed a direct comparison between OFA and OSA while recruiting only cats older than 6 months of age. Additionally, meloxicam was administered preoperatively herein instead of postoperatively. Refinement of the injectable protocol also included reduced doses of ketamine (4 mg/kg instead of 5 or 7 mg/kg) (14). Ketamine may often confound pain assessment since it produces purposeless movement, especially when dexmedetomidine, responsible for sedation, analgesia and muscle relaxation, is antagonized by atipamezole immediately at the end of surgery. Therefore, the refined protocol used in the present study also delayed the administration of atipamezole for 15 min after the end of surgery with the expectations of improved pain management while limiting ketamine-induced adverse effects postoperatively.

As mentioned above, kittens had a lower prevalence of rescue analgesia than adult cats in our previous study using OFA and similar methodology. It demonstrates that age differences could be a confounding factor as the literature indicated that kittens may show fewer behavioral signs of pain than adults or express pain behaviors differently (30). For this reason, this study recruited only cats older than 6 months of age, and therefore, the results should not be extrapolated to kittens. As mentioned before, a recent clinical trial in our laboratory showed that OFA using multimodal analgesia is effective in kittens (26). Our laboratory is currently characterizing pain behaviors and investigating novel anesthetic and analgesic techniques applied specifically to kittens in a different subset of studies, which will be reported in the near future.

The use of KET-DEX-MID has been previously reported as an effective and rapid injectable anesthetic protocol for OVH in cats (14), but it is again worth the discussion as lower doses of ketamine were used in the present study with or without buprenorphine. The onset and duration of anesthesia are adequate to perform OVH (i.e., <20 min) by an experienced surgeon without the need for administration of intraoperative ketamine, and this has been reported with other ketamine-based protocols (31, 32). In terms of high-volume spay-neuter program, the use of an intravenous catheter may not be required with KET-DEX-MID. The duration of anesthesia was hastened by the administration of atipamezole postoperatively. With the exception of “time to lift head”, all other parameters in the anesthetic recovery were similar between groups. Cats in the OFA lifted their heads faster than those in OSA. It is difficult to explain this finding as it could be clinically irrelevant, a result of sedation using buprenorphine in combination with KET-DEX-MID, or that cats in OSA were indeed more comfortable and resting as their FGS scores never increased significantly when compared with baseline. In any case, sedation scores and time to sternal recumbency were not significantly different between groups. Sedation assessment is important in clinical trials as it is unknown how residual anesthetic effects can affect pain assessment using the FGS. As much as sedation may have affected pain scoring in the first 2 h after surgery when scores were increased when compared with baseline, this effect should be similar in both groups as DIVAS scores were not different between groups. Finally, the prevalence of anesthetic complications was low using both OFA and OSA and overall, lower than the previous study using KET-DEX-MID (14). With the exception of desaturation in two cats and tachypnea in four cats, other anesthetic complications were not observed supporting the use of OFA and OSA in clinical practice. Oxygen should be available as one cat with desaturation required supplementation. In the previous study using OFA, desaturation, tachypnea and tachycardia were observed in a large number of cats [7, 4 and 1 cat(s), respectively], but higher doses of ketamine (5 or 7 mg/kg) were administered and could explain these differences in the prevalence of anesthetic complications.

Some limitations of this study have already been presented. For example, it would be interesting to have an additional KET-DEX-MID group without multimodal analgesia with bupivacaine and meloxicam, as this is often the anesthetic protocol used in spay-neuter programs when opioids are not available (3). Additionally, it is not known if other opioids such as methadone or butorphanol would produce the same analgesic effects. The study did not involve any other surgical procedure, male individuals or those with comorbidities making extrapolations difficult to these cases. Finally, results may differ when other ketamine-based protocols are used including doses and drugs such as medetomidine as replacement for dexmedetomidine, or when these latter drugs are not antagonized by atipamezole. When opioids are not available, analgesia produced by agonists of α_2_ adrenergic receptors might become more important and drug antagonism will also reverse analgesia. The current literature supports the reversal of these drugs with minimal effect on analgesia in cats after OVH (31, 33).

In conclusion, opioid-free injectable anesthesia was appropriate for some cats using a multimodal approach including intraperitoneal bupivacaine and preoperative administration of meloxicam. However, single dose IM buprenorphine eliminated the need for rescue analgesia and assured adequate pain management after OVH in cats demonstrating that opioid-sparing techniques still provide optimal analgesia and veterinary care when used as part of multimodal analgesia.

## Data availability statement

The raw data supporting the conclusions of this article will be made available by the authors, without undue reservation.

## Ethics statement

The animal study was reviewed and approved by Animal Care Committee of the Faculty of Veterinary Medicine (FMV), Université de Montréal (20-RECH-2075). Written informed consent was obtained from the owners for the participation of their animals in this study.

## Author contributions

MR, HR, BM, and PS: study design, data collection, data analysis, and manuscript drafting. RW, I-CC, and JB: data collection and manuscript drafting. All authors contributed to the article and approved the submitted version.

## Funding

This study was funded by the Morris Animal Foundation (D22FE-019) including open-access publication fees. The manuscript has not been reviewed or endorsed by the Foundation, and the views expressed do not necessarily reflect the views of the Foundation, its officers, directors, affiliates, or agents. The study received funding from the Fonds du Centenaire of the Faculty of Veterinary Medicine, Université de Montréal. PS laboratory is funded by the National Sciences and Engineering Research Council of Canada (RGPIN-2018-03831).

## Conflict of interest

The authors declare that the research was conducted in the absence of any commercial or financial relationships that could be construed as a potential conflict of interest.

## Publisher's note

All claims expressed in this article are solely those of the authors and do not necessarily represent those of their affiliated organizations, or those of the publisher, the editors and the reviewers. Any product that may be evaluated in this article, or claim that may be made by its manufacturer, is not guaranteed or endorsed by the publisher.
